# Planetary Health Rounds: A novel educational model for integrating healthcare sustainability education into postgraduate medical curricula

**DOI:** 10.1016/j.joclim.2025.100412

**Published:** 2025-01-14

**Authors:** Tajdeep Brar, Jordana Compagnone, Sanjana Sudershan, Maryam Yunus, Loukman Ghouti, Allen Tran, Joffre Munro, Babar Haroon, Nabha Shetty

**Affiliations:** a Dalhousie University Department of Medicine, QEII - Bethune Building, Room 442, VG Site, 1276 South Park Street, Halifax, Nova Scotia, B3H 2Y9, Canada; b Dalhousie University Faculty of Medicine, 1459 Oxford Street, Dalhousie University, Halifax, Nova Scotia, B3H 4R2, Canada; c Division of General Internal Medicine at Dalhousie University Department of Medicine, QEII - Bethune Building, VG Site - 1276 South Park Street, Halifax, Nova Scotia, B3H 2Y9, Canada; d Department of Critical Care at Dalhousie University, QEII – Bethune Building, Suite 377, VG Site – 1276 South Park Street, Halifax, Nova Scotia, B3H 2Y9, Canada

**Keywords:** Healthcare sustainability, Medical education, Climate change education, Health effects of climate change, Life cycle analysis

## Abstract

**Introduction:**

Climate change poses a major threat to public health, necessitating significant reductions in greenhouse gas (GHG) emissions to limit its effects. The healthcare sector itself is a significant contributor to GHG emissions, particularly in high-income countries such as Canada and the United States. Providing medical learners with education on this topic has been identified as an important component of efforts to reduce GHG emissions; however, there is a lack of tools available both for providing education on healthcare sustainability, and for integrating this topic into postgraduate medical curricula.

**Case Presentation:**

The Planetary Health Rounds are an educational initiative aimed at integrating climate change concepts and healthcare sustainability into the Internal Medicine residency curriculum, using a case-analysis format in conjunction with the open-source HealthcareLCA Database (https://healthcarelca.com/database), a living repository of data on healthcare-associated GHG emissions.

**Methods:**

Learners conduct a case analysis of an internal medicine patient and estimate the total emissions associated with their admission, which they then present at an end-of-rotation teaching session, with discussions centering on the link between climate change and health as well as reducing emissions.

**Discussion:**

The Planetary Health Rounds, implemented in 2023, have been well-received by trainee physicians despite some challenges having been encountered. These include service demands impacting participation, a lack of emissions data for internal medicine-related care, issues with the generalizability of said data, and consistent access to a planetary health expertise during rounds.

**Conclusion:**

This initiative provides a novel way of incorporating teaching on climate change and health into postgraduate training curriculums.

## Highlights

• Planetary Health Rounds explore the relationship between climate change and health.
• Planetary Health Rounds provide training on healthcare sustainability practices.
• Emphasis is placed on discussions to reduce emissions as health care practitioners.
• Sustainability principles are similar to Choosing Wisely.

## 1 Introduction

Climate change has been described as the single greatest future hazard to public health in the twenty-first century [1]. However, climate change is already impacting human health around the globe in significant ways. Elevated temperatures, extreme weather events, and worsening air quality are associated with higher morbidity, mortality, and increasing strain on healthcare systems [2]. To avoid the worst effects of climate change, the Paris Climate Agreement currently holds a threshold of limiting the global average temperature rise to less than 2 °Celsius by the end of the century, with a target of 1.5 °Celsius [3]. However, existing greenhouse gas (GHG) emission mitigation efforts are far from sufficient to achieve these goals, with predictions estimating a rise of 3 °Celsius [4,5]. Multiple “tipping points” are at risk of being surpassed, threatening abrupt and/or irreversible damage that further accelerates climate change and its effects [6]. Little research has been done on the eventual “end game” effects of severe and uncontrolled global heating, though the consequences increasingly appear to be catastrophic [7].

Mitigation of the climate crisis through reducing GHG emissions also applies to healthcare, a significant contributor to global GHG emissions. An estimated 5% of global GHG emissions result from healthcare delivery, with significant variation between high- and low-income countries [8,9]. The United States, for example, accounts for approximately one-quarter of all healthcare-related GHG emissions globally, as the sector comprises up to 9-10% of the country's total GHG emissions [10]. Similarly, Canadian healthcare accounts for approximately 4.6% of GHG emissions nationally [8]. Much evidence exists demonstrating the unequal effect globally of the climate crisis, with racialized communities bearing the brunt of climate change's effects [11]. Given North America's disproportionate GHG emissions, both per capita and nationally, compared with the rest of the world, it is important that sectors such as healthcare are addressed [12]. Furthermore, as over a third of healthcare-related emissions are associated with hospital care, hospital-related reductions have the potential for significant impact [13].

Planetary health education and education on climate change and human health have been identified as key components in combatting climate change and limiting its effects on human health [14]. Physicians have previously been shown to be among the most effective advocates for climate change awareness and mitigation [15]. Preparing medical learners in navigating the health impacts of climate change is thus critical for addressing climate change and achieving sustainable health systems, with the need to incorporate education on this topic into medical curricula becoming increasingly acknowledged [15,16,17]. While interested in the topic, medical learners in Canada, the United States, and globally have identified inadequacy in their education on the interplay between climate change and health [18,19,20,21]. One explanation for the discordance between planetary health interest and education is limited access to suitable teaching materials [22]. Even in schools where climate change curricula exists, teaching on healthcare sustainability appears to be a deficit [15,22]. Thus, education on healthcare-associated GHG emissions may represent a particular blind spot in already limited climate change and health curricula, emphasizing the need for tools for healthcare sustainability teaching. To improve access to teaching on climate change and health, as well as to bridge the gap on healthcare sustainability education, we developed a novel teaching tool, the Planetary Health Rounds.

## 2 Case presentation

### 2.1 Conceptualization

The Green Medical Teaching Unit (MTU) group was formed in early 2023 by resident and staff physicians in the Department of Medicine at Dalhousie University in Halifax, Nova Scotia, Canada. The goal was to promote healthcare sustainability and to develop educational programming on Planetary Health for the MTU, the 60-bed general internal medicine inpatient teaching service at the Queen Elizabeth II Health Sciences Center in Halifax. Members of the Green MTU group were introduced to a Canadian project, the HealthcareLCA Database (https://healthcarelca.com/database), through the 2023 CASCADES Summer Institute on Sustainable Health Systems, an annual event providing education on planetary health and healthcare sustainability to graduate trainees in health-related programs [23]. The HealthcareLCA Database is an open-source project based out of the University of Toronto that collates emissions data for medical investigations, treatments, and components of care contained within published Life Cycle Assessments (LCAs) [24]. This database thus provides easily accessible and evidence-based estimates of GHG emissions associated with common healthcare practices.

Though the GHG emissions estimates contained within the HealthcareLCA Database are limited by the unique contexts of the institutions at which they were calculated, and therefore are not readily generalizable, they serve as a pedagogically useful way of presenting and understanding the climate impacts of common healthcare practices. Further, given the utility of LCA's for professionals seeking to assess and reduce GHG emissions, providing trainees with exposure to these tools may benefit future healthcare sustainability efforts [25]. Thus, Green MTU team members sought to utilize the HealthcareLCA Database as a novel means of teaching healthcare sustainability, connecting healthcare actions to real-world climate consequences. This culminated in the Planetary Health Rounds, addressing a gap in climate change-focused teaching within Dalhousie's core Internal Medicine Residency Program.

### 2.2 Format

The Planetary Health Rounds incorporated trainee, or resident, physicians rotating through the MTU during their training programs, both from within and outside of the core Internal Medicine Residency program. Given the success and familiarity of the case-based rounds format used during other MTU teaching sessions such as the Morbidity and Mortality Rounds, a similar approach was selected. Learners conducted chart reviews of patients whose care they had been involved in, cataloguing the number and types of investigations and treatments ordered, and their length of stay in hospital. Collected data was then mapped to corresponding entries in the HealthcareLCA Database, to yield an estimate of the GHG emissions associated with each part of the patient's stay in kilograms of carbon dioxide equivalents (kgCO_2_e). An emissions convertor (the Environmental Protection Agency's Emissions Calculator, https://www.epa.gov/energy/greenhouse-gas-equivalencies-calculator) was then used to convert these values to real world quantities – for example, barrels of oil burnt [26]. At the end of each four-week rotation, this case analysis was presented at the Planetary Health Rounds, attended by all trainee and staff physicians on the MTU, as well as members of the Green MTU team. The goal was to identify areas of potentially outsized GHG emissions, note instances of resource waste, and generate discussion around the link between climate change and healthcare. In order to encourage attendance, rounds were catered with a vegetarian lunch using funding provided by a grant from CASCADES Canada.

### 2.3 Project run

The Planetary Health Rounds were trialed starting in July of 2023, with iterative improvements to timing, format, and involvement of MTU learners. In response to feedback, an orientation to the project with an accompanying mock case was given to each group. In September of 2023 the project was officially launched.

At the start of each four-week MTU rotation, an orientation is held to familiarize learners with concepts of planetary health, healthcare sustainability, and lifecycle analyses, as well as to the objectives of the project. A mock case presentation is done to demonstrate how to conduct the project, as well as to introduce learners to the HealthcareLCA Database. The selection of relatively uncomplicated ‘bread and butter’ cases is recommended to participants to ensure the focus remains on emissions rather than the details of the admission. The limitations in generalizability of GHG emission estimates arising from studies performed in other regions is explained to learners, who are encouraged to consider how local context and power sources impact the estimates. The orientation also emphasizes that the goal of the rounds in highlighting practices that can reduce GHG emissions without sacrificing clinical care.

Post-orientation, participating trainees are tasked with selecting a case, conducting a chart review, tabulating data and then calculating an estimate of the GHG emissions related to the admission using the HealthcareLCA Database. They then convert this estimate into readily comprehendible units using an emissions converter and prepare a presentation. Green MTU team members are available for support throughout this process. On the last Monday of each rotation, Planetary Health Rounds are held in person with a hybrid virtual option available to Green MTU team members and involved learners who are not on-site. Post-case presentation, a facilitated discussion takes place, surrounding strategies to reduce emissions in the future with emphasis on identified areas of outsized emissions from the case that were not necessary for patient care (Table 1).

**Table 1:** Steps for running the Planetary Health Rounds over the course of a four-week rotation.

**Step**	**Tasks**	**Timeline**
1. Orientation	Conduct an orientation session providing an introduction to the relationship between healthcare and climate change, healthcare sustainability, the HealthcareLCA Database, and the purpose of the project, with a mock case to demonstrate how it will work	Week 1
2. Case selection	Assist resident physicians in selecting an appropriate patient case that they have been involved in. Care should be taken to select a patient with a relatively uncomplicated clinical course who will be discharged in the next several days in order to simplify the chart review	Week 2
3. Case review	Assist resident physicians in: • Conducting a chart review and tabulating the number and type of investigations done during the patient's admission, as well as their length of stay • Using the HealthcareLCA Database to obtain estimates of GHG emissions associated with each test, investigation, and day spent in hospital, then using the EPA Emissions Calculator to convert these values into real-world terms • Creating a presentation, which should include an evaluation of potentially avoidable GHG emissions associated with the patient's stay	Week 2–3
4. Planetary Health Rounds presentation	Book a suitable room, arrange catering if funds are available, and ensure participating resident physicians are available for a one-hour period for the Planetary Health Rounds. Then, provide space for the presenting residents to deliver their presentation	Week 4
5. Discussion	Facilitate a group discussion around structured questions, including: • What areas contributed the most to the total emissions for each stay? • What tests/treatments were potentially unnecessary? • How can climate change's local and global impact be mitigated for this patient?	Week 4

## 3 Discussion

### 3.1 Benefits of project

This project allowed for the incorporation of both planetary health and healthcare sustainability education into the MTU curriculum, providing resident physicians from varied backgrounds with the opportunity to learn about these topics to better inform efforts to mitigate climate change – from advocacy to political action and education. It also provided participants with an understanding of LCAs, a valuable tool for those interested in healthcare sustainability. Preliminary results from an ongoing survey of learners perceptions of the utility of the Planetary Health Rounds indicate that learners are finding the project to be beneficial (Table 2). Discussions held during the Planetary Health Rounds were also exceedingly generative. For example, discussions repeatedly and organically linked minimizing emissions with avoiding unnecessary investigations and treatments, a concept that overlaps with initiatives designed to reduce healthcare expenditures while preserving patient care such as the Choosing Wisely campaign [27]. Choosing Wisely's publication of climate-conscious care recommendations this year reinforces this messaging and may provide a scaffolding for future rounds’ discussions [28]. Finally, the project served to generate interest in the link between climate change and health, with emphasis on each learner's areas of interest. For example, one group incorporated information on the spread of zoonotic infections in the local context as a result of climate change, prompting a high-yield discussion of the ramifications of this and possible mitigation strategies.

**Table 2:** Preliminary results of an ongoing efficacy survey of the Planetary Health Rounds from the 2023–2024 academic year.

**Metric**	**Results**
Number of surveys	75
Number of iterations	6
Gained new knowledge as a result of the Rounds	69 (92 % of respondents)
Intend to change practice as a result of the Rounds	62 (83 % of respondents)
General themes of intended changes	Reduce lab work - Stop routine labs - Reconsider reflexive on-call ordering - Focus on physical exam Reduce length of stay - Advocate and support earlier discharge - Earlier transition to PO medications Reduce waste - Decrease paper usage - Wean off supplemental oxygen earlier

### 3.2 Areas for improvement

With increasing demands on our healthcare system, resident physicians on the MTU have high workloads impacting their ability to engage in non-patient care activities such as the Planetary Health Rounds. Additionally, learners have multiple competing educational priorities, often resulting in planetary health teaching being deprioritized in favor of knowledge acquisition directly relevant to the MTU environment. These have both previously been identified as barriers to implementing climate change and health education [16]. The low generalizability of the data from the HealthcareLCA Database also posed an issue, as it necessitated that participants spend extra time comparing the contexts in which LCA studies were done with our own to determine the degree of applicability. While this did promote familiarity with the subject matter, this further added to learner workloads. Several gaps within the HealthcareLCA Database were also identified, including data for less-commonly ordered investigations, making it difficult to make truly comprehensive estimates for patient stays. Given that the database is a living repository, this will likely occur less often in the future.

### 3.3 Future directions

These identified areas have led to several planned adjustments for future iterations. Protected time will be sought for resident physicians on the MTU to participate in the Planetary Health Rounds, to reduce their workload and encourage participation. We are surveying participants in the Planetary Health Rounds to assess their effectiveness and guide future improvements. Additionally, we plan to conduct LCAs for common healthcare practices at our institution to obtain accurate GHG emission estimates that can inform sustainability interventions. Participation in the Planetary Health Rounds has sparked considerable enthusiasm for performing LCAs, accelerating our efforts. There has been interest from other postgraduate training programs in adopting the Planetary Health Rounds, and we along with CASCADES are developing tools to support these initiatives (*Appendix 1*).

## 4 Conclusion

The Planetary Health Rounds format is a novel way of addressing the lack of climate change and health education in postgraduate medical education, and in particular provides a means of teaching fundamental principles of healthcare sustainability to trainee physicians. Given that the project uses an open-source database and freely available online tools, it has the potential to readily be exported to other institutions. Work is ongoing to assess the efficacy of this tool, reduce barriers to learner participation in the project, and provide more comprehensive educational experiences.

## Funding

Funding for this project was provide by a grant from CASCADES, a Canadian capacity-building initiative to address healthcare's contribution to the climate crisis funded by Environment and Climate Change Canada (https://cascadescanada.ca/).

## Glossary

• Climate change – a term describing alterations in global average temperatures as a result of human activities
• Greenhouse Gas (GHG) – gasses that contribute to the greenhouse effect, whereby heat from the sun is prevented from radiating into space and consequently increasing global temperatures, leading to climate change
• Planetary health – the interdependent relationship between the health of the planet and human health, including the effects of climate change on global health
• Healthcare sustainability – an awareness of the environmental impacts of healthcare and its contribution to climate change, with a focus on reducing healthcare-related greenhouse gas emissions
• Medical Teaching Unit (MTU) – a common term reflecting an inpatient internal medicine service in Canada, led by internal medicine specialists and designed to give learners experience with this area of medicine while providing care to medically complex patients. Used interchangeably with Clinical Teaching Unit (CTU), depending on the institution
• Life Cycle Assessment (LCA) – a methodology for assessing the total greenhouse gas emissions associated with an item, from its creation, transportation, usage and disposal
• Kilograms of carbon dioxide equivalents (kgCO_2_e) – the most commonly used unit for quantities of greenhouse gas emissions, allowing the net impact of emissions consisting of multiple greenhouse gasses to be readily relayed

## CRediT authorship contribution statement

**Tajdeep Brar:** Writing – review & editing, Writing – original draft, Project administration, Methodology, Investigation, Formal analysis, Conceptualization. **Jordana Compagnone:** Writing – review & editing, Resources, Project administration, Methodology, Investigation, Conceptualization. **Sanjana Sudershan:** Writing – review & editing, Project administration, Methodology, Investigation, Formal analysis, Conceptualization. **Maryam Yunus:** Writing – review & editing, Project administration, Methodology, Investigation, Conceptualization. **Loukman Ghouti:** Writing – review & editing, Methodology, Data curation, Conceptualization. **Allen Tran:** Supervision, Project administration. **Joffre Munro:** Supervision, Project administration. **Babar Haroon:** Supervision, Project administration. **Nabha Shetty:** Writing – review & editing, Supervision, Project administration, Funding acquisition, Conceptualization.

## Declaration of competing interest

The authors declare that they have no known competing financial interests or personal relationships that could have appeared to influence the work reported in this paper.

## Appendix Supplementary materials

Image, application 1
