# Supplementary material for: Planetary Health Rounds: A novel educational model for integrating healthcare sustainability education into postgraduate medical curricula
Source: J Clim Chang Health. 2025 Jan 14;22:100412. doi: 10.1016/j.joclim.2025.100412 (PMC12851077; doi:10.1016/j.joclim.2025.100412)
Supplement: Supplementary file 1 [file mmc1.pdf]

PLANETARY HEALTH ROUNDS - PREPARATORY PRESENTATION

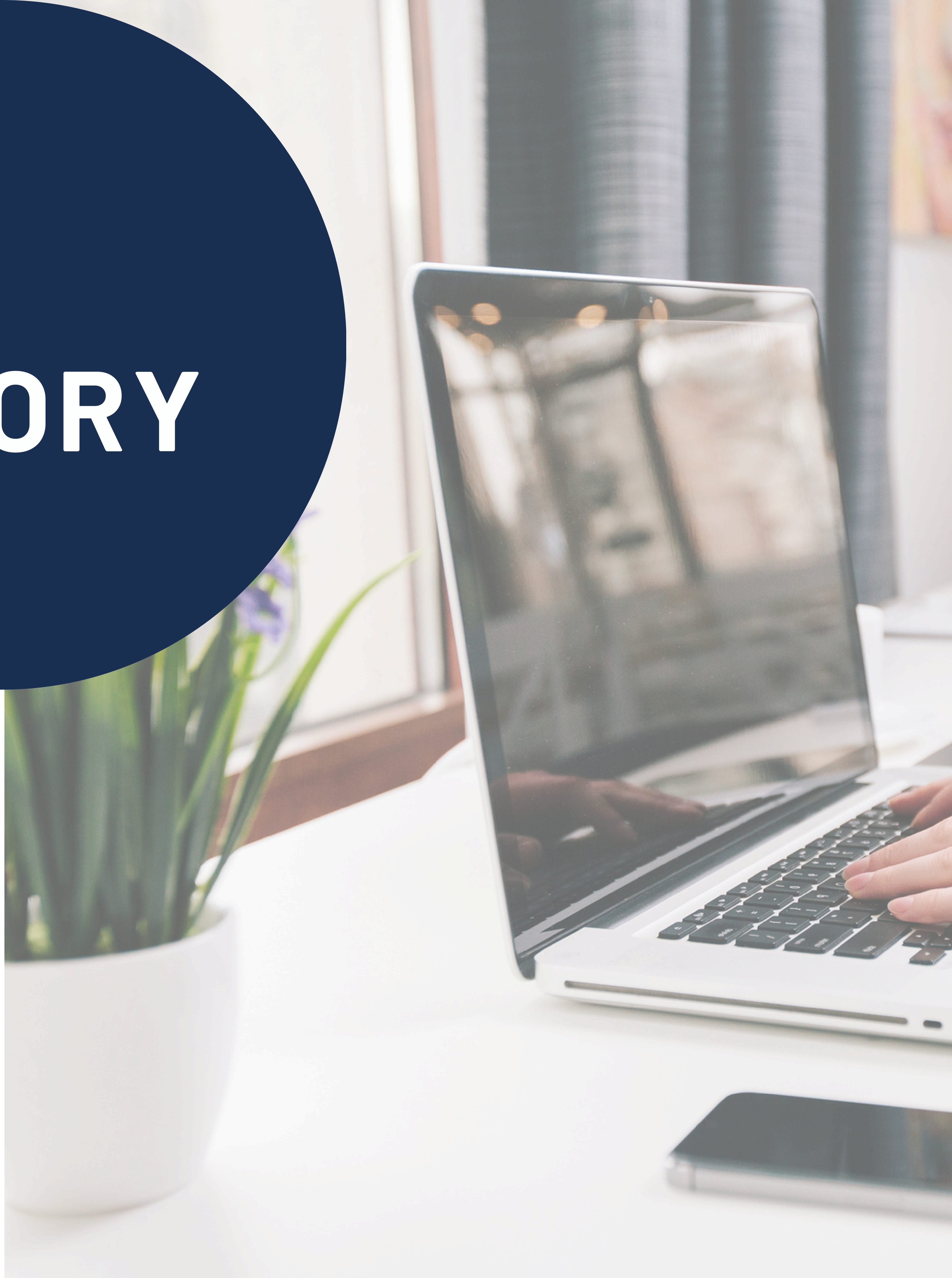

This project was undertaken with the financial support of the Government of Canada.
Ce projet a été réalisé avec l'appui financier du gouvernement du Canada.
Canada

CASCADES

INTRODUCTION

This preparatory slide deck was created to introduce the Planetary Health Rounds format to learners. A Planetary Health Champion, with clinical expertise, will use this deck to explain the intention and format of the Rounds.

This deck provides guidance on what to include in case presentations but does not capture the discussion during Rounds. It is important to have a Planetary Health Champion present during Rounds to provide additional context and to answer questions.

Intention of Planetary Health Rounds

This Rounds format was created so that healthcare teams can better understand how climate change is impacting the health of patients and begin to understand the greenhouse gas (GHG) emissions associated with care. Team members from across health professions can be included in these presentations.

The Planetary Health Rounds presentations identify clinical activities that contributed to GHG emissions but may not have benefited the patient, highlighting opportunities for more sustainable healthcare practice.

These Rounds are intended to provide a starting point for discussions about providing sustainable healthcare. They should be held in addition to other initiatives that raise awareness of planetary health.

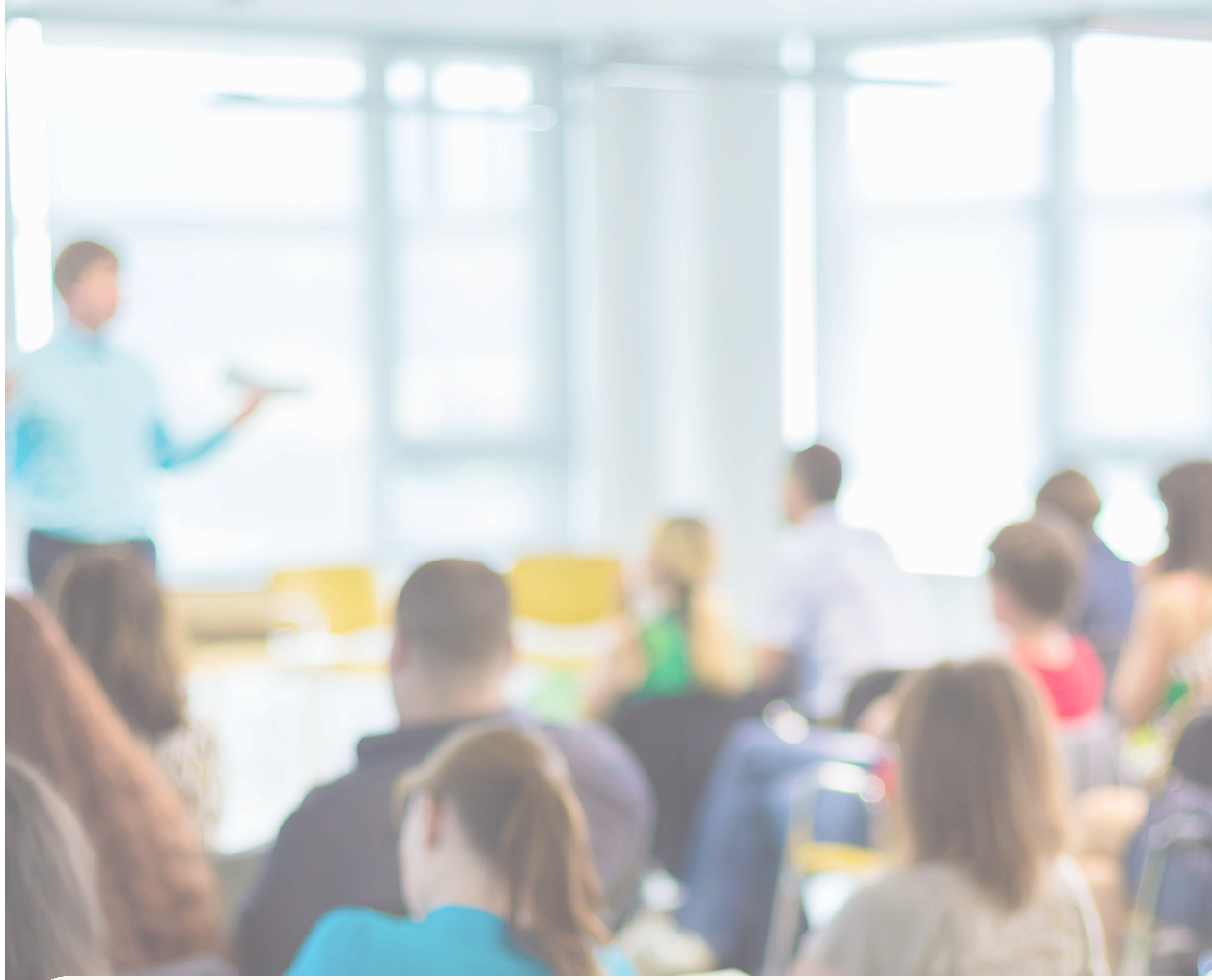

Acknowledgements

This slide deck was developed by the Green MTU team, with the support of CASCADES.

For more information about this project:
https://cascadescanada.ca/resources/planetary-health-education/

Background

UNDERSTANDING GHG EMISSIONS

LIMITATIONS OF USING LCA LITERATURE TO ESTIMATE CO_2E

Published LCA studies are specific to the study context and may not be generalizable to your location. LCA studies use different boundaries to determine what is included in the environmental impact assessment. Factors that may influence environmental impact may not be included (e.g. some LCA studies may account for travel, heating/cooling of the building, or other factors, while other studies do not).

Care must be taken to explain that the values derived are a rough approximation and are not reflective of the actual emissions at your location. These rough estimates can still be used to highlight “hot spots,” but should be considered for informational purposes only.

NOTE TO LEARNERS

Identify a limitation in at least one of the studies you are referencing (e.g. location of study and differences in energy grid, length of diagnostic imaging test, etc.)

UNITS OF MEASUREMENT

Carbon dioxide (CO_2) is the most well-known GHG and the most prevalent in the atmosphere, however, there are multiple greenhouse gases that have different Global Warming Potential (GWP).

GWP is a standardization tool used to compare the global warming impact of different GHGs over a fixed time period. It measures how much energy a gas will absorb compared to an equivalent amount of CO_2.

Carbon dioxide equivalent (CO_2e) is a measure that converts emissions from other GHGs into the equivalent amount of CO_2 using their GWP. CO_2e provides a single number that covers multiple gases.

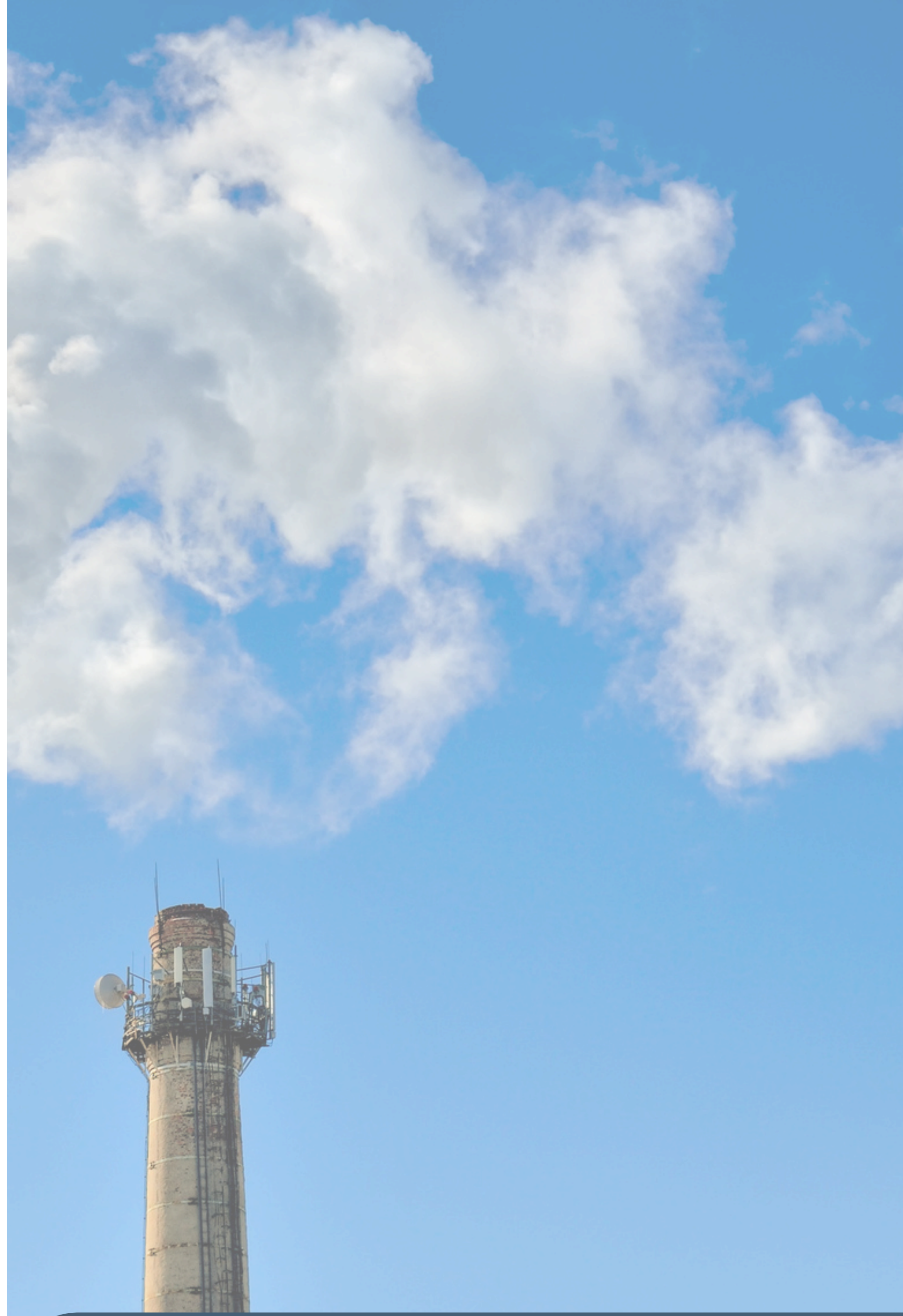

RESOURCES:

For examples of how to estimate the environmental impacts of unnecessary care, as well as information on understanding the limitations of these estimates, see the CASCADES resource Reducing Unnecessary Care: Estimating Environmental Impact

Case Presentation

CREATING THE CASE SUMMARY

Summarize a recently discharged case from your team.

Summary should include:

- Reason for coming to hospital
- Significant medical and social history
- Length of stay in each care area (e.g., emergency department, ward, critical care, transitional care)
- Outcomes of care recevied

PART 1: CLIMATE CHANGE IMPACTS ON HEALTH

Consider the impact that climate change may have had on the patient's health

**Guiding Questions**		
Are there publications that describe the impact of climate change related factors on your patient's medical diagnoses?	How has this person's individual health journey been impacted by exposure or vulnerability to climate change (e.g. – occupational exposures, housing)?	How might recent climate change-related events (e.g. heatwaves, wildfires) have contributed to this hospital admission?

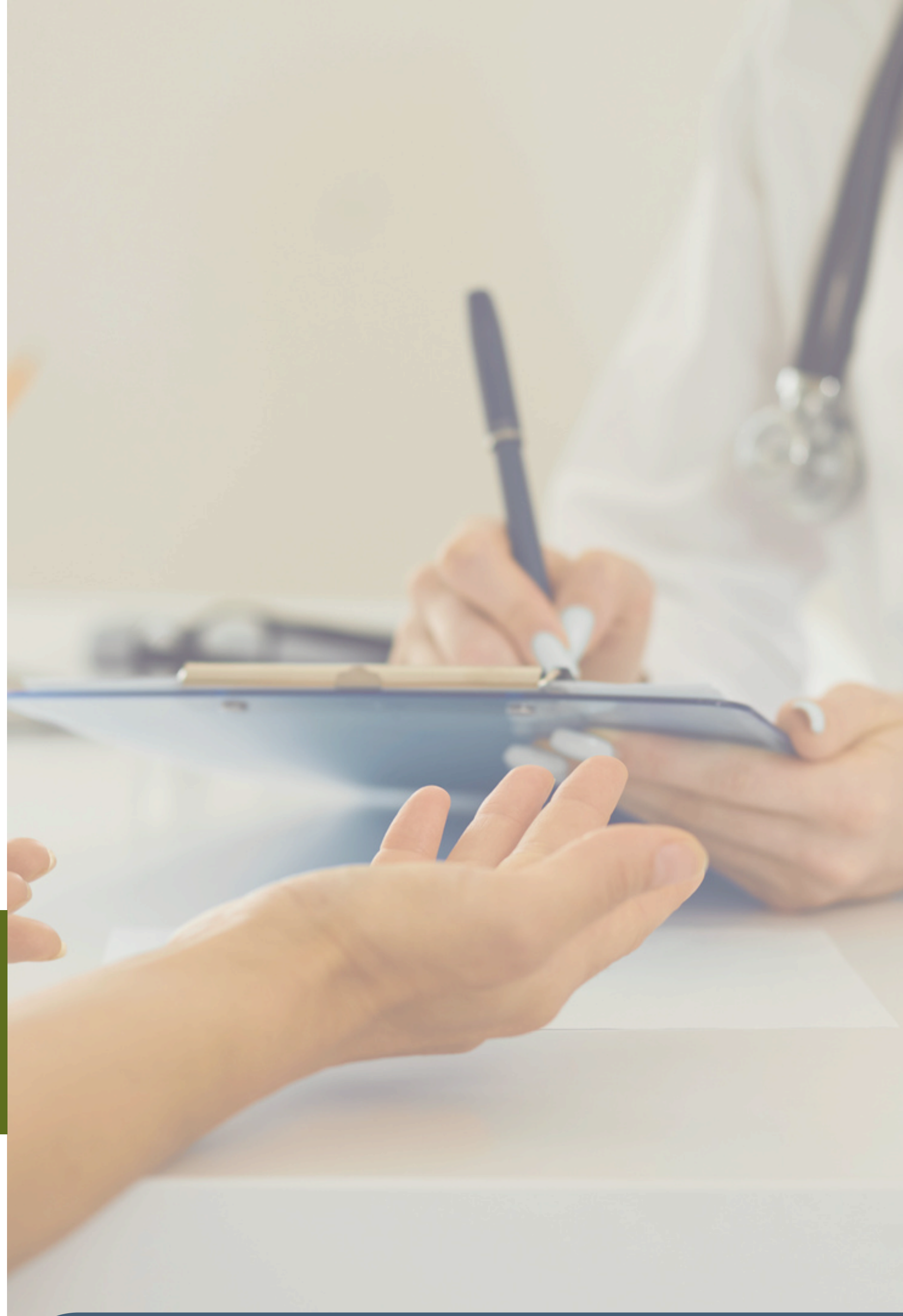

RESOURCES:

The Health of Canadians in a Changing Climate report was released in 2022 and provides an overview of climate change risks to health and the healthcare system.

- The Health of Canadians in a Changing Climate, Health Canada

Case Presentation

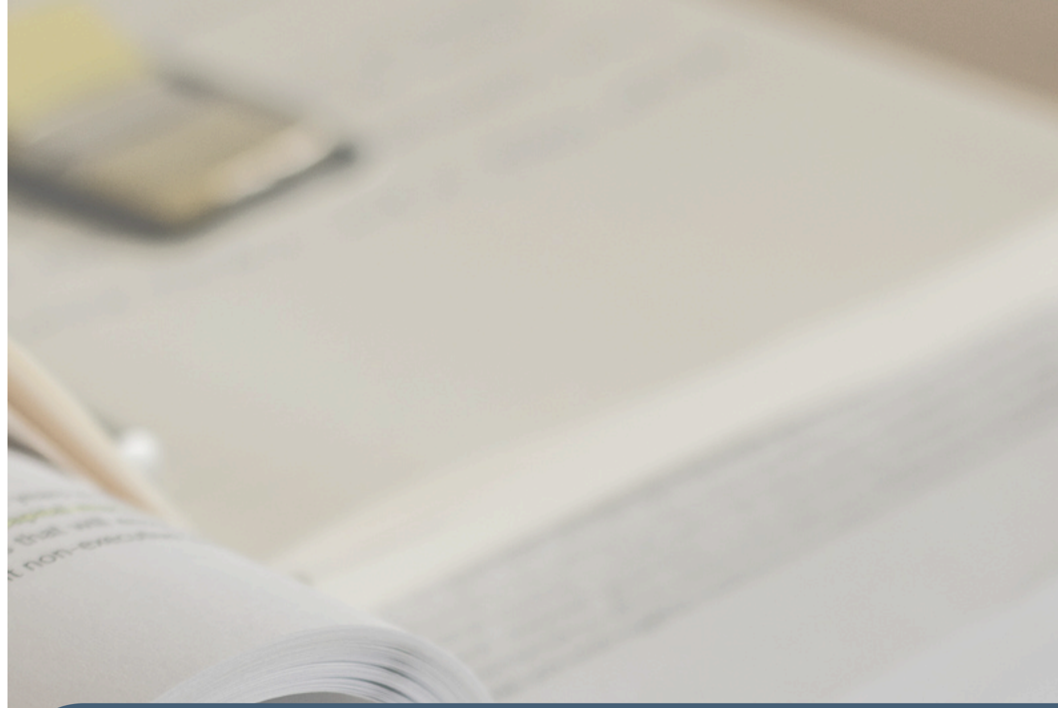

PART 2 - HEALTHCARE’S IMPACT ON GHG EMISSIONS

Conduct a review of all testing and treatment performed during this patient’s hospital stay, broken down by care area (e.g., emergency department, ward, critical care, transitional care).

Find published LCA literature on GHG emissions for the following elements of care:

1. Lab tests
2. Diagnostic imaging
3. Procedures/invasive testing
4. Treatments (pharmacologic and non-pharmacologic)/interventions

You may wish to use HealthcareLCA to find these studies (see right) or look to the grey literature.

Note on boundaries: Determine what emissions sources will be included in the case presentation. You may chose to omit some emissions sources (e.g., transportation to hospital, in-patient food services) for feasibility. Make a note of what is included.

Presenting GHG emissions estimates

Present total estimated emissions per care area by type of test, procedure, and/or intervention. Use pie charts, graphs and other visualizations to make your case. Make sure to include the measurement unit (e.g. KG of CO_2e)

Translate GHG emissions into everyday terms using a tool like Natural Resources Canada’s Greenhouse Gas Equivalencies Calculator or the US-based Environmental Protection Agency’s Greenhouse Gas Equivalencies Calculator. These tools can convert GHG emissions data into everyday units like number of cars on the road. These data can help communicate impact to a broader audience.

RESOURCE:

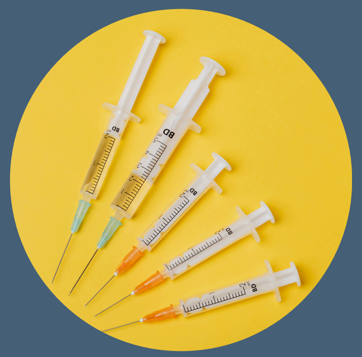

HealthcareLCA is a global living database of healthcare-related environmental impact assessments. Created in collaboration with CASCADES, the database is designed to support the transition to sustainable, low carbon health systems, providing an open-access, interactive, and up-to-date evidence resource for healthcare workers, sustainability researchers, and policy makers. In addition to global warming potential, the HealthcareLCA database includes data sources on a range of environmental impact categories, including eutrophication potential, ozone depletion potential, acidification potential, and others.

Discussion:
Putting it all together

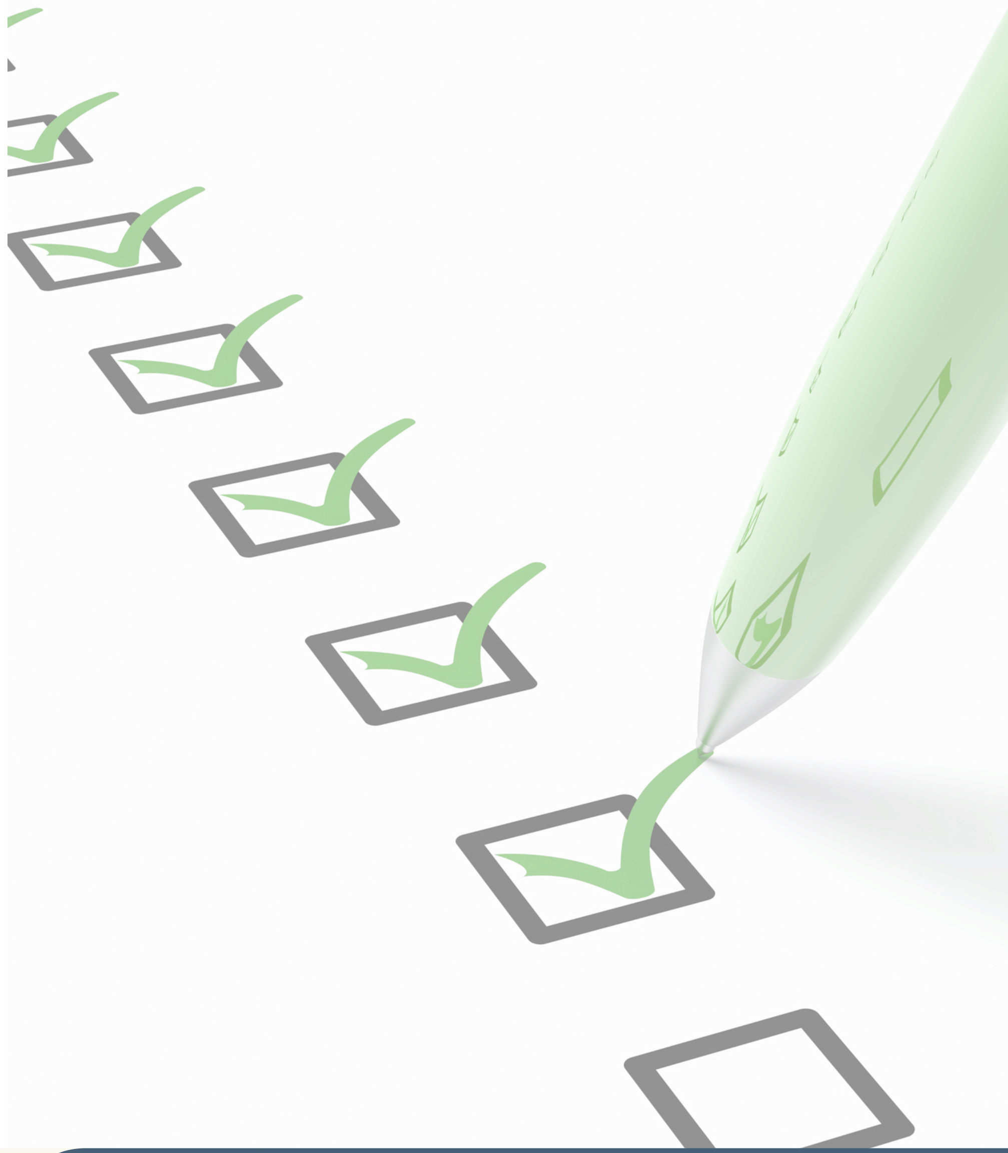

Reflecting on what you have presented, attempt to answer the following questions. The Planetary Health Champion can help with this dicussion during Rounds.

1. How can climate change's impact on health be mitigated for similar patients when it comes to inpatient and outpatient care?
2. What tests/treatments were potentially unnecessary? How could a similar, or better, outcome have been achieved through more environmentally sustainable care?
3. What future "best practices" have been identified from this case review when it comes to planetary health?

HIGH QUALITY, LOW CARBON CARE

These Planetary Health Rounds are not intended to provide or take the place of of clinical guidance. Clinical considerations and following best practices must always come first. The principles of shared decision making with patients should be followed.

RESOURCES:

Choosing Wisely Canada presents numerous recommendations regarding common tests and treatments that are not supported by evidence. These recommendations have been developped by professional societies representing different clinical specialties in Canada.

- Choosing Wisely Canada recommendations by specialty
- Choosing Wisely Canada Climate-Conscious Recommendations
